# *N*-(4-Chloro­phen­yl)pyrrolidine-1-carboxamide

**DOI:** 10.1107/S1600536811024111

**Published:** 2011-06-25

**Authors:** Yu-Feng Li

**Affiliations:** a Microscale Science Institute, Department of Chemistry and Chemical Engineering, Weifang University, Weifang 261061, People’s Republic of China

## Abstract

In the title mol­ecule, C_11_H_13_ClN_2_O, the five-membered ring has an envelope conformation. In the crystal, mol­ecules are linked into chains along [100] by inter­molecular N—H⋯O hydrogen bonds.

## Related literature

For the medicinal properties of pyrrolidine compounds, see: Yang *et al.* (1997 ▶). For a related structure, see: Köhn *et al.* (2004 ▶).
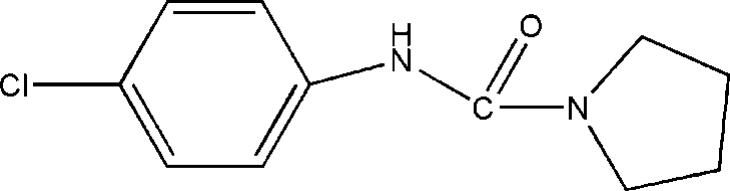


## Experimental

### 

#### Crystal data

C_11_H_13_ClN_2_O
*M* *_r_* = 224.68
Orthorhombic, 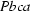

*a* = 9.4498 (19) Å
*b* = 10.856 (2) Å
*c* = 21.930 (4) Å
*V* = 2249.7 (8) Å^3^
*Z* = 8
Mo *K*α radiation
μ = 0.31 mm^−1^
*T* = 293 K
0.23 × 0.19 × 0.19 mm

#### Data collection

Bruker SMART CCD diffractometer
20387 measured reflections
2576 independent reflections
2264 reflections with *I* > 2σ(*I*)
*R*_int_ = 0.036

#### Refinement

*R*[*F* ^2^ > 2σ(*F* ^2^)] = 0.046
*wR*(*F* ^2^) = 0.130
*S* = 1.07
2576 reflections
136 parameters
H-atom parameters constrained
Δρ_max_ = 0.55 e Å^−3^
Δρ_min_ = −0.33 e Å^−3^

### 

Data collection: *SMART* (Bruker, 1997 ▶); cell refinement: *SAINT* (Bruker, 1997 ▶); data reduction: *SAINT*; program(s) used to solve structure: *SHELXS97* (Sheldrick, 2008 ▶); program(s) used to refine structure: *SHELXL97* (Sheldrick, 2008 ▶); molecular graphics: *SHELXTL* (Sheldrick, 2008 ▶); software used to prepare material for publication: *SHELXTL*.

## Supplementary Material

Crystal structure: contains datablock(s) global, I. DOI: 10.1107/S1600536811024111/lh5257sup1.cif

Structure factors: contains datablock(s) I. DOI: 10.1107/S1600536811024111/lh5257Isup2.hkl

Supplementary material file. DOI: 10.1107/S1600536811024111/lh5257Isup3.cml

Additional supplementary materials:  crystallographic information; 3D view; checkCIF report

## Figures and Tables

**Table 1:** Hydrogen-bond geometry (Å, °)

*D*—H⋯*A*	*D*—H	H⋯*A*	*D*⋯*A*	*D*—H⋯*A*
N2—H2*A*⋯O1^i^	0.86	2.21	2.9184 (15)	140

Symmetry code: (i) 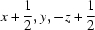.

**Fig. 1.**
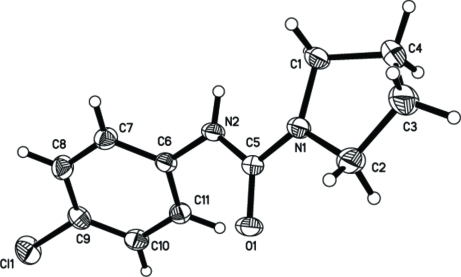
The molecular structure of the title compound showing 30% probability displacement ellipsoids.

## supplementary crystallographic information

### Comment

Pyrrolidine compounds have been shown to have medicinal properties (Yang *et al.*, 1997). The crystal structure of the title compound is presented herein. The molecular structure of the title compound is shown in Fig. 1. The five-membered ring has an envelope conformation with atom C4 forming the flap. In the crystal, the molecules are linked into chains along [100] by intermoecular N—H···O hydrogen bonds. The structure of a related compound has already been determined (Köhn *et al.*, 2004).

### Experimental

A mixture of pyrrolidine (0.1 mol), and (4-chlorophenyl)carbamic chloride (0.1 mol) was stirred in refluxing ethanol (20 ml) for 4 h to afford the title compound (0.079 mol, yield 79%). Colourless blocks of the title compound were obtained by recrystallization of a solution of the title compound ethanol at room temperature.

### Refinement

H atoms were fixed geometrically and allowed to ride on their attached atoms, with C—H distances = 0.93–0.97 Å; N—H = 0.86Å and with *U*_iso_(H) = 1.2*U*_eq_(C,*N*).

### Crystal data

**Table:**

C_11_H_13_ClN_2_O	*F*(000) = 944
*M**_r_* = 224.68	*D*_x_ = 1.327 Mg m^−^^3^
Orthorhombic, *P**b**c**a*	Mo *K*α radiation, λ = 0.71073 Å
Hall symbol: -P 2ac 2ab	Cell parameters from 2264 reflections
*a* = 9.4498 (19) Å	θ = 3.0–27.6°
*b* = 10.856 (2) Å	µ = 0.31 mm^−^^1^
*c* = 21.930 (4) Å	*T* = 293 K
*V* = 2249.7 (8) Å^3^	Bar, colorless
*Z* = 8	0.23 × 0.19 × 0.19 mm

### Data collection

**Table:**

Bruker SMART CCD diffractometer	2264 reflections with *I* > 2σ(*I*)
Radiation source: fine-focus sealed tube	*R*_int_ = 0.036
graphite	θ_max_ = 27.5°, θ_min_ = 3.0°
φ and ω scans	*h* = −12→10
20387 measured reflections	*k* = −14→14
2576 independent reflections	*l* = −28→28

### Refinement

**Table:**

Refinement on *F*^2^	Primary atom site location: structure-invariant direct methods
Least-squares matrix: full	Secondary atom site location: difference Fourier map
*R*[*F*^2^ > 2σ(*F*^2^)] = 0.046	Hydrogen site location: inferred from neighbouring sites
*wR*(*F*^2^) = 0.130	H-atom parameters constrained
*S* = 1.07	*w* = 1/[σ^2^(*F*_o_^2^) + (0.0763*P*)^2^ + 0.5404*P*] where *P* = (*F*_o_^2^ + 2*F*_c_^2^)/3
2576 reflections	(Δ/σ)_max_ < 0.001
136 parameters	Δρ_max_ = 0.55 e Å^−^^3^
0 restraints	Δρ_min_ = −0.33 e Å^−^^3^

### Special details

**Table:**

Geometry. All e.s.d.'s (except the e.s.d. in the dihedral angle between two l.s. planes) are estimated using the full covariance matrix. The cell e.s.d.'s are taken into account individually in the estimation of e.s.d.'s in distances, angles and torsion angles; correlations between e.s.d.'s in cell parameters are only used when they are defined by crystal symmetry. An approximate (isotropic) treatment of cell e.s.d.'s is used for estimating e.s.d.'s involving l.s. planes.
Refinement. Refinement of *F*^2^ against ALL reflections. The weighted *R*-factor *wR* and goodness of fit *S* are based on *F*^2^, conventional *R*-factors *R* are based on *F*, with *F* set to zero for negative *F*^2^. The threshold expression of *F*^2^ > σ(*F*^2^) is used only for calculating *R*-factors(gt) *etc*. and is not relevant to the choice of reflections for refinement. *R*-factors based on *F*^2^ are statistically about twice as large as those based on *F*, and *R*- factors based on ALL data will be even larger.

### Fractional atomic coordinates and isotropic or equivalent isotropic displacement parameters (Å^2^)

**Table:**

	*x*	*y*	*z*	*U*_iso_*/*U*_eq_	
Cl1	0.10254 (6)	0.10302 (5)	0.03994 (2)	0.0685 (2)	
O1	0.12968 (10)	0.43109 (12)	0.28838 (5)	0.0496 (3)	
N2	0.34453 (11)	0.36694 (12)	0.25166 (5)	0.0388 (3)	
H2A	0.4347	0.3673	0.2570	0.047*	
C6	0.28726 (13)	0.30130 (12)	0.20175 (6)	0.0347 (3)	
C10	0.12280 (16)	0.15354 (14)	0.16052 (7)	0.0434 (3)	
H10A	0.0507	0.0963	0.1660	0.052*	
C5	0.26031 (14)	0.43003 (13)	0.29184 (6)	0.0356 (3)	
C11	0.17977 (15)	0.21521 (13)	0.20997 (6)	0.0393 (3)	
H11A	0.1460	0.1990	0.2490	0.047*	
N1	0.32890 (12)	0.49261 (12)	0.33577 (5)	0.0417 (3)	
C9	0.17459 (17)	0.17824 (14)	0.10291 (7)	0.0445 (3)	
C7	0.34090 (16)	0.32181 (14)	0.14377 (7)	0.0420 (3)	
H7A	0.4150	0.3770	0.1383	0.050*	
C1	0.47839 (15)	0.47967 (16)	0.35253 (7)	0.0463 (4)	
H1A	0.5367	0.5390	0.3310	0.056*	
H1B	0.5127	0.3973	0.3439	0.056*	
C8	0.28451 (17)	0.26042 (15)	0.09408 (7)	0.0460 (4)	
H8A	0.3202	0.2743	0.0552	0.055*	
C2	0.24919 (18)	0.5611 (2)	0.38171 (8)	0.0584 (5)	
H2B	0.1790	0.5091	0.4012	0.070*	
H2C	0.2023	0.6319	0.3638	0.070*	
C4	0.4771 (2)	0.5051 (2)	0.42042 (8)	0.0633 (5)	
H4A	0.4543	0.4313	0.4434	0.076*	
H4B	0.5677	0.5367	0.4341	0.076*	
C3	0.3624 (2)	0.6009 (2)	0.42688 (10)	0.0748 (7)	
H3A	0.3981	0.6822	0.4169	0.090*	
H3B	0.3253	0.6021	0.4681	0.090*	

### Atomic displacement parameters (Å^2^)

**Table:**

	*U*^11^	*U*^22^	*U*^33^	*U*^12^	*U*^13^	*U*^23^
Cl1	0.0781 (4)	0.0754 (4)	0.0520 (3)	−0.0222 (2)	−0.0144 (2)	−0.0182 (2)
O1	0.0252 (5)	0.0734 (8)	0.0503 (6)	−0.0018 (5)	−0.0022 (4)	−0.0141 (5)
N2	0.0250 (5)	0.0529 (7)	0.0385 (6)	−0.0035 (4)	−0.0019 (5)	−0.0104 (5)
C6	0.0300 (6)	0.0374 (7)	0.0366 (6)	−0.0003 (5)	−0.0028 (5)	−0.0035 (5)
C10	0.0401 (7)	0.0376 (7)	0.0525 (8)	−0.0078 (6)	−0.0040 (6)	−0.0022 (6)
C5	0.0273 (6)	0.0462 (7)	0.0333 (6)	−0.0018 (5)	−0.0008 (5)	−0.0012 (5)
C11	0.0378 (7)	0.0399 (7)	0.0403 (7)	−0.0038 (5)	−0.0005 (5)	0.0017 (5)
N1	0.0278 (6)	0.0607 (8)	0.0366 (6)	0.0020 (5)	−0.0021 (5)	−0.0122 (5)
C9	0.0476 (8)	0.0423 (7)	0.0435 (7)	−0.0040 (6)	−0.0103 (6)	−0.0082 (6)
C7	0.0407 (7)	0.0434 (7)	0.0420 (7)	−0.0097 (6)	0.0024 (6)	−0.0037 (6)
C1	0.0304 (7)	0.0660 (9)	0.0427 (7)	0.0017 (6)	−0.0066 (6)	−0.0109 (7)
C8	0.0516 (8)	0.0499 (8)	0.0366 (7)	−0.0080 (6)	0.0007 (6)	−0.0041 (6)
C2	0.0386 (7)	0.0854 (12)	0.0511 (9)	0.0037 (8)	0.0039 (7)	−0.0269 (9)
C4	0.0507 (10)	0.0972 (14)	0.0419 (8)	−0.0043 (9)	−0.0111 (7)	−0.0109 (8)
C3	0.0550 (10)	0.1115 (18)	0.0580 (11)	−0.0022 (11)	−0.0013 (9)	−0.0434 (11)

### Geometric parameters (Å, °)

**Table:**

Cl1—C9	1.7428 (15)	C7—C8	1.384 (2)
O1—C5	1.2368 (17)	C7—H7A	0.9300
N2—C5	1.3707 (18)	C1—C4	1.514 (2)
N2—C6	1.4138 (16)	C1—H1A	0.9700
N2—H2A	0.8600	C1—H1B	0.9700
C6—C7	1.387 (2)	C8—H8A	0.9300
C6—C11	1.3920 (19)	C2—C3	1.521 (3)
C10—C9	1.381 (2)	C2—H2B	0.9700
C10—C11	1.383 (2)	C2—H2C	0.9700
C10—H10A	0.9300	C4—C3	1.509 (3)
C5—N1	1.3453 (17)	C4—H4A	0.9700
C11—H11A	0.9300	C4—H4B	0.9700
N1—C2	1.4612 (19)	C3—H3A	0.9700
N1—C1	1.4664 (18)	C3—H3B	0.9700
C9—C8	1.383 (2)		
			
C5—N2—C6	121.82 (11)	C4—C1—H1A	111.2
C5—N2—H2A	119.1	N1—C1—H1B	111.2
C6—N2—H2A	119.1	C4—C1—H1B	111.2
C7—C6—C11	119.55 (12)	H1A—C1—H1B	109.1
C7—C6—N2	119.27 (12)	C9—C8—C7	119.31 (14)
C11—C6—N2	121.17 (12)	C9—C8—H8A	120.3
C9—C10—C11	119.02 (13)	C7—C8—H8A	120.3
C9—C10—H10A	120.5	N1—C2—C3	103.37 (14)
C11—C10—H10A	120.5	N1—C2—H2B	111.1
O1—C5—N1	121.34 (13)	C3—C2—H2B	111.1
O1—C5—N2	123.01 (12)	N1—C2—H2C	111.1
N1—C5—N2	115.65 (12)	C3—C2—H2C	111.1
C10—C11—C6	120.49 (13)	H2B—C2—H2C	109.1
C10—C11—H11A	119.8	C3—C4—C1	102.93 (15)
C6—C11—H11A	119.8	C3—C4—H4A	111.2
C5—N1—C2	120.15 (12)	C1—C4—H4A	111.2
C5—N1—C1	126.53 (12)	C3—C4—H4B	111.2
C2—N1—C1	111.87 (12)	C1—C4—H4B	111.2
C10—C9—C8	121.30 (13)	H4A—C4—H4B	109.1
C10—C9—Cl1	119.70 (12)	C4—C3—C2	104.35 (15)
C8—C9—Cl1	118.99 (12)	C4—C3—H3A	110.9
C8—C7—C6	120.25 (13)	C2—C3—H3A	110.9
C8—C7—H7A	119.9	C4—C3—H3B	110.9
C6—C7—H7A	119.9	C2—C3—H3B	110.9
N1—C1—C4	102.78 (13)	H3A—C3—H3B	108.9
N1—C1—H1A	111.2		

### Hydrogen-bond geometry (Å, °)

**Table:**

*D*—H···*A*	*D*—H	H···*A*	*D*···*A*	*D*—H···*A*
N2—H2A···O1^i^	0.86	2.21	2.9184 (15)	140

Symmetry codes: (i) *x*+1/2, *y*, −*z*+1/2.
